# Diagnostic accuracy of two-dimensional shear wave elastography and point shear wave elastography in identifying different stages of liver fibrosis in patients with metabolic dysfunction-associated steatotic liver disease: A meta-analysis

**DOI:** 10.17305/bb.2024.11577

**Published:** 2025-01-10

**Authors:** Xiangyi Xu, Yiqing Zhang, Qiwei Zhu, Yuchen Xie, Yuanyuan Zhou, Bingtian Dong, Chaoxue Zhang

**Affiliations:** 1 Department of Ultrasound, The First Affiliated Hospital of Anhui Medical University, Hefei, Anhui, China

**Keywords:** Two-dimensional shear wave elastography, 2-D SWE, point shear wave elastography, pSWE, metabolic dysfunction-associated steatotic liver disease, MASLD, liver fibrosis

## Abstract

To assess the diagnostic accuracy of two-dimensional shear wave elastography (2-D SWE) and point shear wave elastography (pSWE) in detecting liver fibrosis stages in patients with metabolic dysfunction-associated steatotic liver disease (MASLD), a comprehensive search was conducted across four databases up to February 9, 2024. A bivariate random-effects model was used to analyze the diagnostic accuracy of the methods. After screening, 13 studies involving pSWE included 1527 patients, while nine studies involving 2-D SWE included 1088 patients. The areas under the summary receiver operating characteristic (SROC) curves for diagnosing significant fibrosis (*F* ≥ 2), advanced fibrosis (*F* ≥ 3), and cirrhosis (*F* ═ 4) using pSWE and 2-D SWE were as follows: 0.84 (95% CI 0.80–0.87), 0.91 (95% CI 0.88–0.93), and 0.94 (95% CI 0.91–0.95) for pSWE; 0.83 (95% CI 0.79–0.86) 0.85 (95% CI 0.82–0.88), and 0.89 (95% CI 0.86–0.91) for 2-D SWE, respectively. The pooled sensitivity for pSWE and 2-D SWE for stages *F* ≥ 2, *F* ≥ 3, and *F* ═ 4 were 0.71 (95% CI 0.63–0.78), 0.81 (95% CI 0.72–0.88), and 0.81 (95% CI 0.63–0.91) for pSWE, and 0.77 (95% CI 0.68–0.84), 0.80 (95% CI 0.72–0.87), and 0.92 (95% CI 0.75–0.98) for 2-D SWE, respectively. The pooled specificity of pSWE and 2-D SWE for these stages were 0.83 (95% CI 0.76–0.88), 0.87 (95% Cl: 0.81–0.92), and 0.91 (95% CI 0.86–0.94) for pSWE, and 0.76 (95% CI 0.66–0.84), 0.76 (95% CI 0.69–0.82), and 0.83 (95% CI 0.78–0.85) for 2-D SWE, respectively. In conclusion, both 2-D SWE and pSWE demonstrated high diagnostic performance in identifying various stages of liver fibrosis in MASLD patients.

## Introduction

It is estimated that roughly 30% of the global population is affected by metabolic dysfunction-associated steatotic liver disease (MASLD), previously known as non-alcoholic fatty liver disease (NAFLD). MASLD is a leading cause of chronic liver disease, impacting approximately 25% of adults worldwide. As the prevalence of diabetes and obesity continues to rise, the burden of MASLD is projected to increase significantly [1, 2]. By 2030, the prevalence is expected to rise by 18%, and by 2040, it could increase by approximately 50% [3–7]. MASLD often begins asymptomatically but may progress to more severe conditions, including metabolic dysfunction-associated steatohepatitis (MASH), liver fibrosis, and cirrhosis. Furthermore, some MASLD patients may develop sarcopenia [3, 8–10]. These complications not only result in direct liver damage but also have far-reaching effects on overall health, quality of life, mental well-being, and economic stability. Liver fibrosis is a key determinant of long-term outcomes for MASLD patients and a significant predictor of mortality in this population [11, 12]. Therefore, accurate fibrosis staging is critical for effective management. Although liver biopsy remains the gold standard for diagnosing and staging fibrosis [13], it is invasive, subject to sampling errors, and carries risks, such as bleeding and infection [14, 15]. Additionally, the high cost of liver biopsies imposes further strain on healthcare systems. In recent years, research has increasingly focused on non-invasive methods for evaluating liver fibrosis, aiming to reduce reliance on biopsies. One study found that shear wave elastography (SWE)-based strategies were the most cost-effective for diagnosing fibrosis stage *F* ≥ 2, while the combination of FIB-4 and SWE was most effective and economical for stage *F* ≥ 3. These findings suggest that broad implementation of risk stratification programs using non-invasive testing is a cost-effective approach to identifying significant fibrosis in MASLD patients [16]. The two most commonly employed non-invasive methods for liver fibrosis evaluation are serum biomarkers and imaging techniques. Serum biomarkers, such as the fibrosis-4 index (FIB-4) and NAFLD fibrosis score (NFS), are widely accessible, simple to use, and cost-efficient. However, their diagnostic accuracy can be influenced by various factors and may vary across different populations [17]. Among imaging techniques, ultrasound elastography is the predominant modality for liver fibrosis assessment. This includes point shear wave elastography (pSWE), transient elastography (TE), and two-dimensional shear wave elastography (2-D SWE). While TE is the most commonly used imaging method, it has limitations, particularly in patients with significant obesity or narrow intercostal spaces, which may require special probes for adequate assessment. SWE, on the other hand, measures liver stiffness indirectly by quantifying shear wave speed variations within tissues, which correlate with the degree of fibrosis. Both pSWE and 2-D SWE use conventional grayscale ultrasound imaging paired with acoustic radiation force to induce tissue deformation. Shear waves generated by this deformation are detected using ultrasound plane wave technology to assess tissue stiffness [18]. While pSWE relies on a fixed region of interest (ROI) measuring 5 × 10 mm to capture single-point data [19], 2-D SWE provides real-time, quantitative elasticity maps of liver tissue over a broader area, allowing operators to adjust the ROI size as needed prior to measurement [20–23]. These features enable both modalities to integrate seamlessly into conventional ultrasound systems and avoid interference from anatomical structures, such as blood vessels, ribs, or bile ducts. Numerous studies have demonstrated the high diagnostic performance of both pSWE and 2-D SWE in evaluating liver fibrosis in MASLD patients, with low rates of measurement failure [24–27]. A 2020 meta-analysis by Zhou et al. [28] found that both techniques exhibited high sensitivity and specificity for detecting liver fibrosis across various stages. Notably, 2-D SWE demonstrated greater sensitivity than pSWE in identifying significant and advanced fibrosis. However, most studies included in the meta-analysis assessed liver fibrosis across multiple liver diseases, with fewer specifically focusing on MASLD. To address this gap, we aim to conduct a meta-analysis to comprehensively evaluate the diagnostic performance of pSWE and 2-D SWE in identifying significant fibrosis, advanced fibrosis, and cirrhosis specifically in MASLD patients. This analysis will synthesize data from previously published studies employing these modalities for liver fibrosis staging in this population.

## Materials and methods

This report rigorously adhered to the Preferred Reporting Items for a Systematic Review and Meta-analysis of Diagnostic Test Accuracy (PRISMA-DTA) guidelines [29]. Furthermore, the study protocol was registered with PROSPERO under the ID CRD 42024550381.

### Study design and search strategies

To investigate the diagnostic performance of these two modalities in detecting liver fibrosis among MASLD patients, two researchers conducted comprehensive searches in PubMed, Cochrane, Web of Science, and Embase, covering all records up to February 19, 2024. The searches utilized a combination of MeSH and free-text terms, including “metabolic dysfunction-associated steatotic liver disease,” “MASLD,” “non-alcoholic fatty liver disease,” “NAFLD,” “2-D shear wave elastography,” “2-D SWE,” “point shear wave elastography,” “Acoustic Radiation Force Impulse,” “ARFI,” and “pSWE,” as well as related expressions. To ensure thoroughness, the references of relevant literature were also screened to avoid omitting any studies. Details of the search strategy are provided in the Supplementary material.

### Study eligibility

The inclusion criteria were as follows: (1) Studies that evaluated the diagnostic performance of 2-D SWE or pSWE for assessing liver fibrosis in MASLD patients. (2) Studies that utilized liver biopsy as the reference standard, employing the Nonalcoholic Steatohepatitis Clinical Research Network (NASH-CRN) system, where *F* ≥ 2 denotes significant fibrosis, *F* ≥ 3 denotes advanced fibrosis, and *F* ═ 4 denotes cirrhosis. (3) Studies that provided data on sensitivity and specificity, as well as the number of patients across various fibrosis stages, enabling the construction of at least one 2×2 contingency table based on true/false positive and true/false negative rates. (4) Publications written in English. The exclusion criteria wereas follows: (1) Studies that did not provide diagnostic performance data for 2-D SWE or pSWE. (2) Studies that included patients with chronic liver diseasesother than MASLD. (3) Studies with incomplete data. (4) Reviews, meta-analyses, animal experiments, conference abstracts, guidelines, or letters.

### Data extraction and screening of the literature

The obtained publications were independently screened by two researchers using predetermined inclusion and exclusion criteria. Duplicate studies were initially removed using Endnote X9 software (Clarivate Analytics). Next, the remaining studies underwent title and abstract screening to exclude irrelevant ones. The full texts of preliminarily included studies were then thoroughly reviewed for final selection. Screening results were cross-validated, and any discrepancies were resolved through discussions involving a third researcher. Two reviewers independently extracted data from the studies, including author names, publication year, study period, country, evaluation system, patient count, mean age, gender distribution, sensitivity, and specificity. Any missing data in the original articles were recorded as “not reported.” If disagreements arose during data extraction, a third researcher was consulted to facilitate resolution.

### Study quality assessment

The quality and risk of bias in the included studies were evaluated using the QUADAS-2 tool [30]. This assessment framework considered criteria, such as patient selection, index test, reference standard, and flow and timing. Applicability concerns were also assessed regarding the reference standard, index test, and patient selection. Two researchers conducted the quality assessments independently and cross-checked their findings. Any disputes were resolved through discussion with a third researcher. The RevMan 5.4 software (The Cochrane Community, London, UK) was used to visually represent the quality of the included studies.

### Statistical analysis

Taking into consideration the classifications of *F* ≥ 2, *F* ≥ 3, and *F* ═ 4, 2×2 contingency tables were developed to facilitate the analysis. The primary objective of the study was to evaluate the diagnostic accuracy of 2-D SWE and pSWE in identifying *F* ≥ 2 fibrosis. In secondary analyses, the researchers assessed the methods’ ability to identify *F* ≥ 3 fibrosis and cirrhosis (*F* ═ 4).

All obtained diagnostic data were analyzed using the MIDAS module of Meta-Disc Statistical Software, Version 1.4 (Unit of Clinical Biostatistics, Ramon y Cajal Hospital, Madrid, Spain) and Stata15.0 (StataCorp, College Station, TX, USA), employing a bivariate mixed-effects model. Reliable statistical metrics are produced by the model, which takes into account variables like sample size, threshold effect, and inter-study heterogeneity while retaining the original data’s bivariate structure throughout the analysis. For the purpose of calculating the synthesized sensitivity and specificity data, as well as the positive likelihood ratio (PLR), the negative likelihood ratio (NLR), the diagnostic odds ratio (DOR), and diagnostic score (DS), forest maps were subsequently plotted. Higher DS and DOR values signify superior diagnostic performance. The area under the curve (AUC) was derived from the summary receiver operating characteristic (SROC) curve. Based on AUC of 0.5–0.7, 0.7–0.9, and 0.9–1.0, respectively, diagnostic efficacy was classified as poor, moderate, or high. Sensitivity analysis was used to evaluate how each study affected the overall findings and to check if the summary statistics were stable. The Spearman correlation coefficient and corresponding *P* value were used to determine whether a threshold effect was present. *P* > 0.05 denoted the absence of threshold effect heterogeneity across the research. Higgins’ *I*^2^ statistics and Cochran’s *Q* test were used to quantify heterogeneity. If there was significant heterogeneity between the studies, as indicated by a *P* less than 0.10 or an *I*^2^ greater than 50%, a fixed-effects model would be used instead of a random-effects model, and vice versa. In the event that a significant degree of heterogeneity was discovered, meta-regression and subgroup analyses would be executed in order to examine the most significant sources of heterogeneity.

The subgroup analysis primarily focused on five dimensions: the region of the enrolled patients (Asia vs non-Asia), the system used (Aixplorer vs non-Aixplorer), sex (male proportion >50% vs male proportion <50%), clinical setting (multicenter vs single-center), and population characteristics (obese vs non-obese). The funnel plot developed by Deeks was utilized to assess the presence of publication bias, with a significance level of *P* < 0.05 being considered significant.

## Results

### Literature search

**Figure 1.**
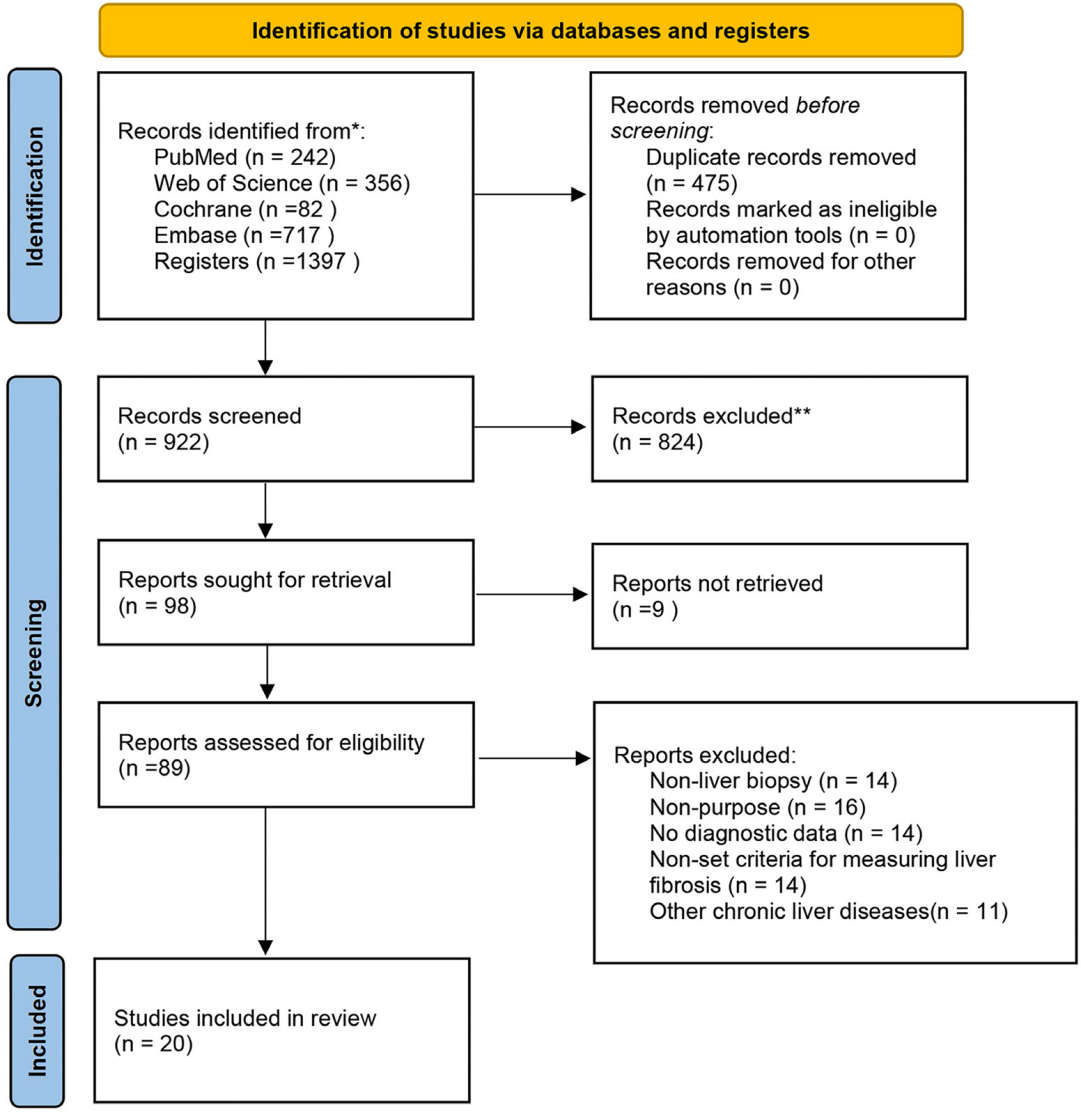
Flow diagram of the study selection process.

As of February 19, 2024, we initially identified 1397 potential articles for inclusion. After removing 475 duplicates, 922 articles proceeded to title and abstract screening, during which 824 were excluded. Following full-text review, 69 additional articles were excluded for the following reasons: (1) lack of diagnostic data for 2×2 contingency table extraction (*n* ═ 14); (2) absence of histology as the gold standard (*n* ═ 14); (3) lack of standardized liver fibrosis measurement criteria (*n* ═ 14); (4) presence of other chronic liver diseases (*n* ═ 11); and (5) absence of liver fibrosis staging detection using pSWE or 2-D SWE (*n* ═ 16). Ultimately, 20 studies met the inclusion criteria, with nine reporting on 2-D SWE and 13 on pSWE. The selection process is illustrated in Figure 1.

### Study features

A total of 1527 patients were included in 13 studies on p-SWE, while 1088 patients were included in nine studies on 2-D SWE. Table 1 summarizes the key features of the studies on 2-D SWE and p-SWE. The failure rates for the two testing methods ranged from 0% to 43% for p-SWE and 0% to 27% for 2-D SWE, respectively. Notably, 31% of the articles did not report p-SWE-related failure rates, while 22% did not report 2-D SWE-related failure rates, as detailed in the Supplementary material.

### Evaluation of quality

Assessed using the QUADAS-2 tool, most studies demonstrated a low overall risk of bias. However, 30% reported an unclear risk of bias related to patient selection. Regarding the index test, 40% of studies showed an unclear risk, while 45% exhibited a high risk of bias. For the reference standard, two studies had a considerable risk of bias, and one study had an uncertain risk. In terms of timing and flow, 55.6% of the data had a high risk of bias, and 30% presented an unclear risk. Despite these concerns, all studies showed minimal risk in the applicability domains of patient selection, index test, and reference standard (Figure S1).

### Meta-analysis

#### Significant liver fibrosis (F≥2)

**Figure 2.**
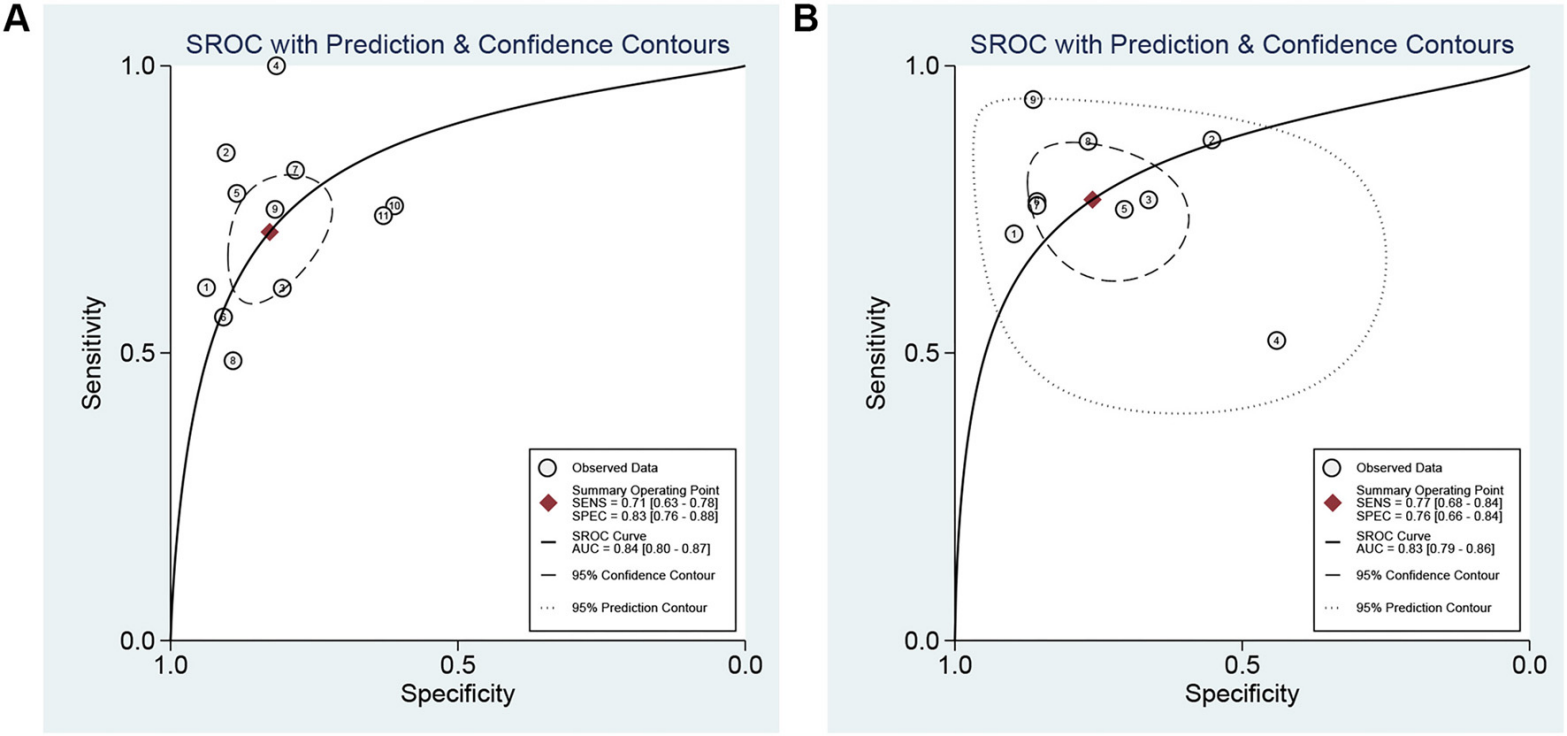
**The area under the ROC curve for the diagnosis of significant fibrosis (*F* ≥ 2).** (A) pSWE; (B) 2-D SWE. pSWE: Point shear wave elastography; 2-D SWE: Two-dimensional shear wave elastography.

The diagnostic accuracy of significant liver fibrosis (*F* ≥ 2) detection using pSWE was analyzed based on 11 studies. The Spearman correlation coefficient, along with its corresponding *P* value (*P* ═ 0.401), indicated no heterogeneity among studies due to a threshold effect. The synthesized sensitivity and specificity were 0.71 (95% CI: 0.63–0.78) and 0.83 (95% CI: 0.76–0.88), respectively (Figure S2). The PLR, NLR, and DOR were 4.12 (95% CI: 2.97–5.72), 0.35 (95% CI: 0.27–0.45), and 11.78 (95% CI: 7.50–18.49), respectively. The SROC curve AUC was 0.84 (95% CI: 0.80–0.87; Figure 2A). Similarly, data for 2-D SWE were extracted from nine studies. The Spearman correlation coefficient and its corresponding *P* value (*P* ═ 0.814) again indicated no heterogeneity among studies due to a threshold effect. The pooled sensitivity and specificity were calculated to be 0.77 (95% CI: 0.68–0.84) and 0.76 (95% CI: 0.66–0.84), respectively (Figure S3). The synthesized PLR, NLR, and DOR were 3.20 (95% CI: 2.12–4.85), 0.31 (95% CI: 0.21–0.45), and 10.45 (95% CI: 5.00–21.84), respectively. The SROC AUC was 0.83 (95% CI: 0.79–0.86; Figure 2B).

**Table 1:** Study characteristics

**Author**	**Year**	**Type**		**Country**	**Sample size (male(%))**	**Age**	**Clinical setting**	**System used**	**Cutoff values** **(m/s)**	**SE**	**Sp**
		**2d**	**P**								
Jamialahmadi	2019	yes		Iran	90 (20)	38.5	Singlecentre	Aixplorer	*F* ≥ 26.6 *F* ≥ 36.75 *F* ═ 46.75	0.75 0.80 1.00	0.71 0.72 0.71
Argalia	2022		yes	Italy	50 (64)	52.2 ± 13.0	Singlecentre	ElastPQ	*F* ≥ 24.63 kPa *F* ≥ 37.39 kPa *F* ═ 414.2 kPa	0.74 0.88 1.00	0.63 0.88 1.00
Attia	2016		yes	Germany	97 (52)	50 ± 12	Singlecentre	Acuson S2000	*F* ≥ 21.18 *F* ≥ 31.47 *F* ═ 41.89	0.78 0.94 0.86	0.88 0.97 0.94
Cassinotto	2013		yes	France	60 (67)	56 ± 13	Singlecentre	Acuson S2001	*F* ≥ 11.18 *F* ≥ 21.81 *F* ≥ 32.03 F ═ 42.54	0.49 0.61 0.65 0.68	1.00 0.94 0.79 0.93
Cassinotto	2016		yes	France	291 (59.1)	56.7 ± 12	Multicentre	Acuson S2000	*F* ≥ 21.32 *F* ≥ 31.53 *F* ═ 42.04	0.56 0.59 0.44	0.91 0.90 0.90
		yes						Aixplorer	*F* ≥ 28.7 kPa *F* ≥ 310.7kPa *F* ═ 414.4 kPa	0.71 0.71 0.58	0.90 0.90 0.90
Cui	2016		yes	USA	125 (45.6)	48.9 ± 15.4	Singlecentre	Acuson S3000	*F* ≥ 21.34 *F* ≥ 31.34 *F* ═ 42.48	0.82 0.95 0.78	0.78 0.74 0.93
Fierbinteanu	2013		yes	Romania	64 (42.9)	48.5	Singlecentre	Acuson S2000	*F* ≥ 21.17 *F* ≥ 31.48 *F* ═ 41.64	0.85 0.86 0.92	0.90 0.95 0.92
Joo	2018		yes	Korea	315 (51)	55 ± 17.78	Singlecentre	Acuson S2000	*F* ≥ 31.40	0.71	0.92
Karlas	2015		yes	Germany	89 (41.6)	50.9 ± 11.4	Singlecentre	Acuson S2000	*F* ≥ 21.25	1	0.82
Lee	2017		yes	Korea	83 (44)	56 ± 13	Singlecentre	Acuson S2000	*F* ≥ 21.29 *F* ≥ 31.36 *F* ═ 41.5	0.49 0.91 0.75	0.9 0.90 0.91
		yes						Aixplorer	*F* ≥ 28.30 kPa *F* ≥ 310.70 kPa *F* ═ 415.10 kPa	0.87 0.90 0.90	0.55 0.61 0.78
Leong	2020		yes	Malaysia	100 (46)	57.1	Singlecentre	Phillips EPIQ 7	*F* ≥ 26.98 *F* ≥ 37.02 *F* ═ 411.52	0.76 0.76 0.75	0.61 0.58 0.93
Medellin	2019		yes	Canada	51	NA	Singlecentre	Acuson S3000	*F* ≥ 21.34 *F* ≥ 31.55 *F* ═ 41.8	0.75 0.90 1.00	0.82 0.77 0.68
Ogino	2023	yes		Japan	107 (60.75)	51 ± 14	Singlecentre	LOGIQ® E9	*F* ≥ 21.47 *F* ≥ 31.58 *F* ═ 41.68	0.87 0.78 0.92	0.77 0.79 0.82
Ozturk	2020	yes		USA	116 (47)	51 ± 12	Singlecentre	Aixplorer	*F* ≥ 21.67 *F* ≥ 31.76	0.77 0.84	0.66 0.7
Palmeri	2011		yes	USA	135 (51)	NA	Singlecentre	Siemen, Sonoline Antares	*F* ≥ 34.24 kPa	0.9	0.9
Seo	2023	yes		Korea	105 (50.5)	36 ± 16.67	Singlecentre	Aplio i800	*F* ≥ 27.1 kPa *F* ≥ 37.7 kPa *F* ═ 48.8 kPa	0.94 0.92 0.80	0.86 0.84 0.85
Sharpton	2021	yes		USA	114 (45.6)	55 ± 4.75	Singlecentre	Aixplorer	*F* ≥ 27.7 kPa *F* ≥ 37.7 kPa *F* ═ 49.3 kPa	0.76 0.90 0.89	0.86 0.78 0.85
Sugimoto	2020	yes		Japan	111 (51.3)	53 ± 18	Singlecentre	Aplio i800	*F* ≥ 21.4 *F* ≥ 31.4 *F* ═ 41.55	0.76 0.85 1.00	0.86 0.79 0.82
Takeuchi	2018	yes		Japan	71 (65)	51 ± 16	Singlecentre	Aixplorer	*F* ≥ 211.57 kPa *F* ≥ 313.07 kPa *F* ═ 415.7 kPa	0.52 0.63 1.00	0.44 0.56 0.82
Zhang	2014		yes	China	67 (69)	34.7 ± 13.2	Singlecentre	Acuson S2000	*F* ≥ 21.31 *F* ≥ 31.36 *F* ═ 41.36	0.61 0.69 1.00	0.81 0.82 0.75

2-D SWE: Two-dimensional shear wave elastography; pSWE: Point shear wave elastography; SE: Sensitivity; Sp: Specificity.

#### Advanced liver fibrosis (F ≥ 3)

**Figure 3.**
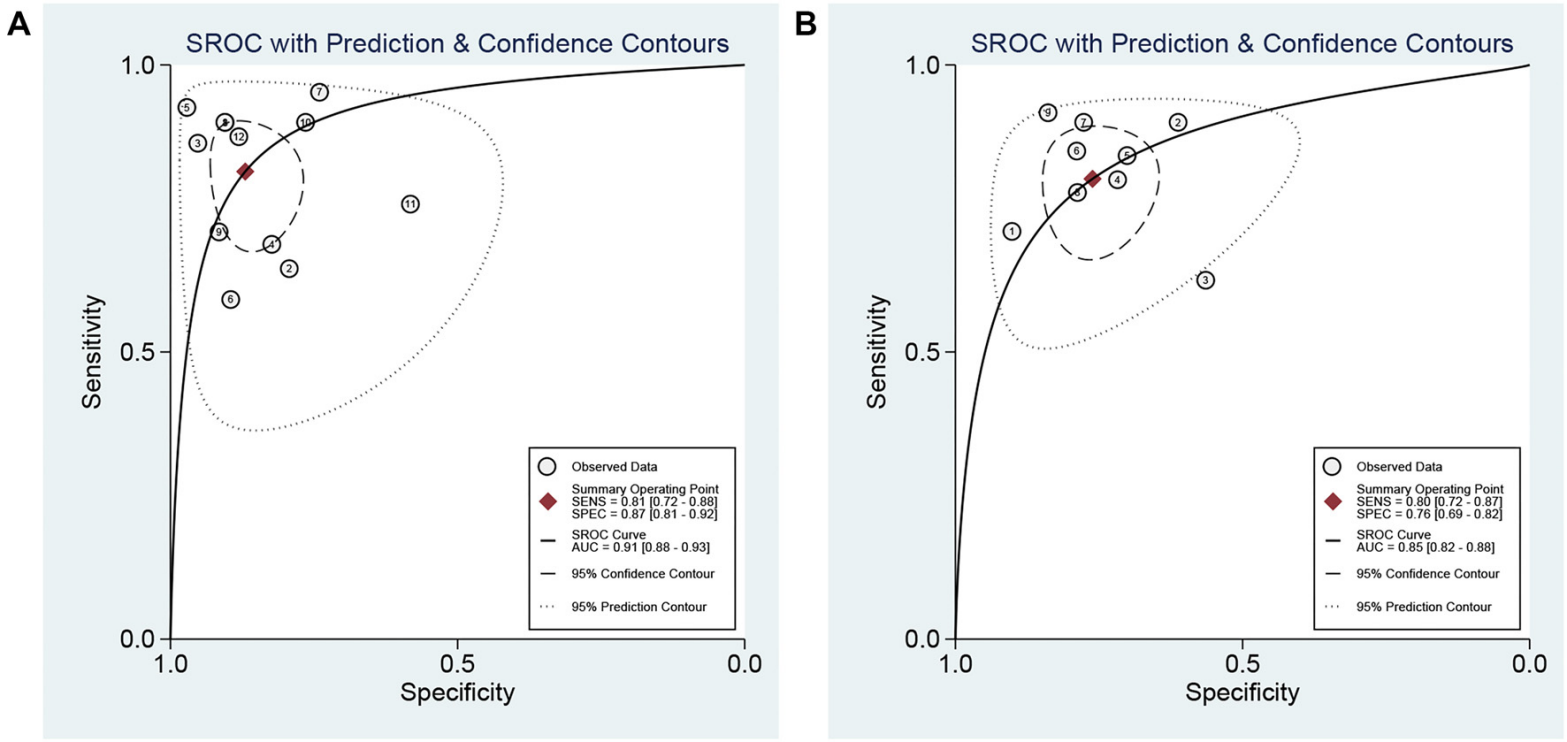
**The area under the ROC curve for the diagnosis of advanced fibrosis (*F* ≥ 3).** (A) pSWE; (B) 2-D SWE. pSWE: Point shear wave elastography; 2-D SWE: Two-dimensional shear wave elastography.

The data on pSWE were collected from 12 studies. The Spearman correlation coefficient (*P* ═ 0.794) for threshold effect identification suggested no heterogeneity among studies due to the threshold effect. The pooled sensitivity and specificity were 0.81 (95% CI: 0.72–0.88) and 0.87 (95% CI: 0.81–0.92), respectively (Figure S4). Additionally, PLR, NLR, and DOR were 6.25 (95% CI: 4.03–9.70), 0.21 (95% CI: 0.14–0.33), and 29.27 (95% CI: 13.86–61.81), respectively. The associated SROC AUC was 0.91 (95% CI: 0.88–0.93; Figure 3A). The results for the diagnostic accuracy of 2-D SWE in detecting advanced liver fibrosis (*F* ≥ 3) were based on data from nine studies. The Spearman correlation coefficient (*P* ═ 0.683) for threshold effect identification indicated no heterogeneity among studies due to the threshold effect. The pooled sensitivity and specificity were 0.80 (95% CI: 0.72–0.87) and 0.76 (95% CI: 0.69–0.82), respectively (Figure S5). The pooled PLR, NLR, and DOR were 3.36 (95% CI: 2.53–4.46), 0.26 (95% CI: 0.18–0.38), and 12.87 (95% CI: 17.25–22.85), respectively. The associated SROC AUC was 0.85 (95% CI: 0.82–0.88; Figure 3B).

#### Cirrhosis (F ═ 4)

**Figure 4.**
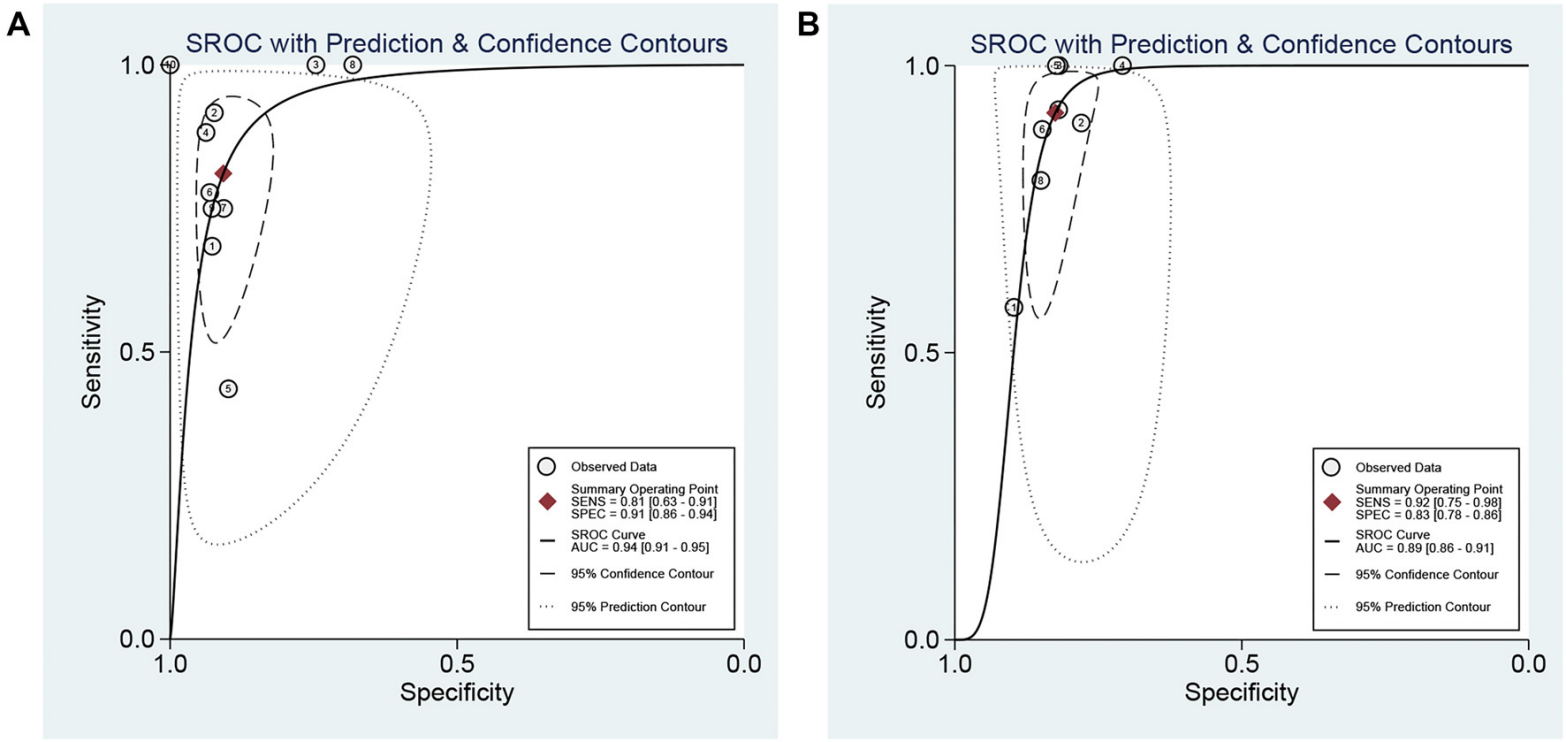
**The area under the ROC curve for the diagnosis of cirrhosis (*F* ═ 4).** (A) pSWE; (B) 2-D SWE. pSWE: Point shear wave elastography; 2-D SWE: Two-dimensional shear wave elastography.

The data on pSWE were collected from 10 studies. The Spearman correlation coefficient and its corresponding *P* value (*P* ═ 0.662) for threshold effect identification indicated no heterogeneity among the studies due to the threshold effect. The synthesized sensitivity and specificity were 0.81 (95% CI: 0.63–0.91) and 0.91 (95% CI: 0.86–0.94), respectively (Figure S6). The synthesized PLR, NLR, and DOR were 8.75 (95% CI: 5.58–13.73), 0.21 (95% CI: 0.10–0.43), and 42.00 (95% CI: 16.35–107.85), respectively. The associated SROC AUC was 0.94 (95% CI: 0.91–0.95) (Figure 4A). The results on the diagnostic accuracy of 2-D SWE for cirrhosis (*F* ═ 4) were derived from eight studies. The Spearman correlation coefficient and its corresponding *P* value (*P* ═ 0.531) for threshold effect identification also indicated no heterogeneity among the studies due to the threshold effect. The synthesized sensitivity and specificity were 0.92 (95% CI: 0.75–0.98) and 0.83 (95% CI: 0.78–0.85), respectively (Figure S7). The synthesized PLR, NLR, and DOR were 5.24 (95% CI: 4.27–6.44), 0.10 (95% CI: 0.03–0.33), and 52.62 (95% CI: 15.34–180.49), respectively. The associated SROC AUC was 0.89 (95% CI: 0.86–0.91) (Figure 4B).

#### Subgroup analyses for 2-D SWE studies

Subgroup analyses based on various covariates were conducted to identify potential sources of heterogeneity: region (Asia vs non-Asia), system used (Aixplorer f vs non-Aixplorer f), sex (male proportion >50% vs male proportion <50%), and clinical setting (multicenter vs single-center) (Table 2). Subgroup quantitative analysis of 2-D SWE based on different regions indicated that, across all stages (*F* ≥ 2, *F* ≥ 3, and *F* ═ 4), diagnostic specificity in Asian regions was lower compared to non-Asian regions. Another subgroup analysis based on the system used revealed that at the *F* ≥ 2 stage, the SEN and SPE of studies using the Aixplorer device were 0.73 [0.63–0.84] and 0.67 [0.57–0.77], respectively, which were lower than the SEN and SPE values of 0.80 [0.70–0.90] and 0.85 [0.78–0.92] reported for other devices. Similarly, at the *F* ≥ 3 stage, the SPE for studies using the Aixplorer device was 0.69 [0.63–0.76], compared to 0.84 [0.79–0.89] for studies using other devices. At the *F* ═ 4 stage, the SPE for studies using the Aixplorer device was 0.79 [0.73–0.85], which was also lower than the SPE of 0.85 [0.81–0.89] for studies utilizing other devices. Subgroup quantitative analysis of 2-D SWE based on sex demonstrated that at stages *F* ≥ 2 and *F* ≥ 3, diagnostic sensitivity was lower in populations with a male proportion >50% compared to those with a male proportion <50%. Additionally, subgroup analysis based on clinical setting showed that at stages *F* ≥ 3 and *F* ═ 4, diagnostic sensitivity in multicenter settings was lower than in single-center settings. Lastly, at the *F* ═ 4 stage, SPE in obese populations was 0.78 [0.70–0.87], which was lower than the SPE of 0.84 [0.80–0.88] in non-obese populations.

**Table 2:** Subgroup analysis of 2-D SWE

**Stages**	**Factors**		**Number of studies**	**Pooled sensitivity**	***P* value**	**Pooled specificity**	***P* value**
*F* ≥ 2	Country	Asia	6	0.79 [0.70–0.88]	0.32	0.72 [0.61–0.84]	**<0.05**
		Non-Asia	3	0.73 [0.60–0.86]		0.82 [0.70–0.94]	
	System used	Aixplorer f	5	0.73 [0.63–0.84]	**0.02**	0.67 [0.57–0.77]	**<0.001**
		Non-Aixplorer f	4	0.80 [0.70–0.90]		0.85 [0.78–0.92]	
	Sex	Proportion of males >50%	5	0.74 [0.65–0.84]	**0.03**	0.80 [0.70–0.90]	0.6
		Proportion of males <50%	4	0.79 [0.69–0.90]		0.71 [0.57–0.85]	
*F* ≥ 3	Country	Asia	6	0.81 [0.72–0.89]	0.15	0.73 [0.65–0.82]	**<0.01**
		Non-Asia	3	0.79 [0.67–0.91]		0.81 [0.72–0.90]	
	System used	Aixplorer f	5	0.81 [0.71–0.91]	0.13	0.69 [0.63–0.76]	**<0.001**
		Non-Aixplorer f	4	0.79 [0.70–0.89]		0.84 [0.79–0.89]	
	Sex	Proportion of males >50%	5	0.75 [0.67–0.82]	**<0.001**	0.80 [0.73–0.87]	0.16
		Proportion of males <50%	4	0.88 [0.79–0.96]		0.71 [0.62–0.80]	
	Clinical setting	Multicentre	1	0.71 [0.55–0.87]	**0.02**	0.90 [0.83–0.97]	0.92
		Singlecentre	8	0.82 [0.75–0.89]		0.74 [0.68–0.79]	
*F* ═ 4	Country	Asia	6	0.94 [0.88–1.00]	0.06	0.80 [0.76–0.84]	**<0.001**
		Non-Asia	2	0.69 [0.46–0.92]		0.88 [0.83–0.92]	
	System used	Aixplorer f	4	0.95 [0.86–1.00]	0.14	0.79 [0.73–0.85]	**<0.001**
		Non-Aixplorer f	4	0.87 [0.70–1.00]		0.85 [0.81–0.89]	
	Clinical setting	Multicentre	1	0.58 [0.42–0.74]	**<0.001**	0.90 [0.85–0.94]	**<0.01**
		Singlecentre	7	0.93 [0.87–1.00]		0.81 [0.78–0.84]	
	Population characteristic	Obesity	2	0.96 [0.85–1.00]	0.06	0.78 [0.70–0.87]	**<0.001**
		Non-obesity	6	0.90 [0.78–1.00]		0.84 [0.80–0.88]	

*Note*: *F* ≥ 2: Significant liver fibrosis; *F* ≥ 3: Advanced liver fibrosis; *F* ═ 4: Cirrhosis; 2-D SWE: Two-dimensional shear wave elastography.

#### Subgroup analyses for pSWE studies

Subgroup analyses based on different covariates were conducted to explore potential sources of heterogeneity: region (Asia vs non-Asia), system used (Acuson vs non-Acuson), sex (male proportion >50% vs male proportion <50%), and clinical setting (multicenter vs single center) (Table 3). Subgroup quantitative analysis of pSWE based on regions demonstrated that, across all stages (*F* ≥ 2, *F* ≥ 3, and *F* ═ 4), the diagnostic specificity in Asian regions was inferior to that in non-Asian regions. Additionally, at the *F* ≥ 2 stage, the diagnostic sensitivity in Asian regions was lower than in non-Asian regions. A subgroup analysis related to the system used indicated that, at the *F* ═ 4 stage, the SPE for studies using the Acuson device was 0.89 [0.84–0.94], which was lower than the specificity of 0.96 [0.92–1.00] for studies using other devices. Subgroup quantitative analysis of 2-D SWE based on sex showed that, at stages *F* ≥ 2 and *F* ≥ 3, the diagnostic sensitivity was lower in populations with a male proportion >50% compared to those with a male proportion <50%. At the *F* ═ 4 stage, the diagnostic specificity was lower in populations with a male proportion >50% compared to those with a male proportion <50%. Subgroup quantitative analysis of pSWE based on clinical settings demonstrated that, across all stages (*F* ≥ 2, *F* ≥ 3, and *F* ═ 4), the diagnostic sensitivity in multicenter settings was lower than in single-center settings.

**Table 3:** Subgroup analysis of pSWE

**Stages**	**Factors**		**Number of studies**	**Pooled sensitivity**	***P* value**	**Pooled specificity**	***P* value**
*F* ≥ 2	Country	Asia	3	0.63 [0.47–0.78]	**0.03**	0.78 [0.66–0.90]	**0.01**
		Non-Asia	8	0.74 [0.66–0.83]		0.84 [0.78–0.91]	
	Sex	Proportion of males >50%	5	0.66 [0.56–0.77]	**0.02**	0.85 [0.77–0.93]	0.13
		Proportion of males <50%	5	0.76 [0.65–0.86]		0.81 [0.71–0.90]	
	Clinical setting	Multicentre	1	0.56 [0.37–0.75]	**0.04**	0.91 [0.81–1.00]	0.92
		Singlecentre	10	0.73 [0.66–0.80]		0.81 [0.75–0.88]	
*F* ≥ 3	Country	Asia	4	0.77 [0.63–0.91]	0.05	0.84 [0.73–0.94]	**0.01**
		Non-Asia	8	0.84 [0.75–0.92]		0.89 [0.83–0.94]	
	Sex	Proportion of males >50%	7	0.75 [0.65–0.86]	**0.01**	0.90 [0.85–0.95]	0.36
		Proportion of males <50%	4	0.88 [0.79–0.97]		0.81 [0.70–0.92]	
	Clinical setting	Multicentre	1	0.59 [0.36–0.83]	**0.01**	0.90 [0.76–1.00]	0.92
		Singlecentre	11	0.83 [0.76–0.89]		0.87 [0.81–0.92]	
*F* ═ 4	Country	Asia	3	0.85 [0.63–1.00]	0.68	0.88 [0.79–0.96]	**<0.01**
		Non-Asia	7	0.79 [0.63–0.95]		0.92 [0.88–0.96]	
	System used	Acuson	8	0.81 [0.66–0.97]	0.82	0.89 [0.84–0.94]	**<0.01**
		Non-Acuson	2	0.86 [0.53–1.00]		0.96 [0.92–1.00]	
	Clinical setting	Multicentre	1	0.44 [0.17–0.70]	**0.01**	0.90 [0.78–1.00]	0.39
		Singlecentre	9	0.84 [0.74–0.94]		0.91 [0.87–0.95]	
	Sex	Proportion of males >50%	5	0.73 [0.54–0.92]	0.3	0.91 [0.86–0.96]	**<0.01**
		Proportion of males <50%	4	0.83 [0.66–0.99]		0.93 [0.88–0.97]	

*Note*: *F* ≥ 2: Significant liver fibrosis; *F* ≥ 3: Advanced liver fibrosis; *F* ═ 4: Cirrhosis; pSWE: Point shear wave elastography.

### Significance comparison between pSWE and 2-D SWE

Upon comparison, no significant difference was observed in the synthesized sensitivity of 2-D SWE and pSWE for identifying significant fibrosis (*P* ═ 0.46). Similarly, there was no notable difference in their ability to detect advanced fibrosis (*P* ═ 0.45) or cirrhosis (*P* ═ 0.369) regarding synthesized sensitivity.

### Publication bias

**Figure 5.**
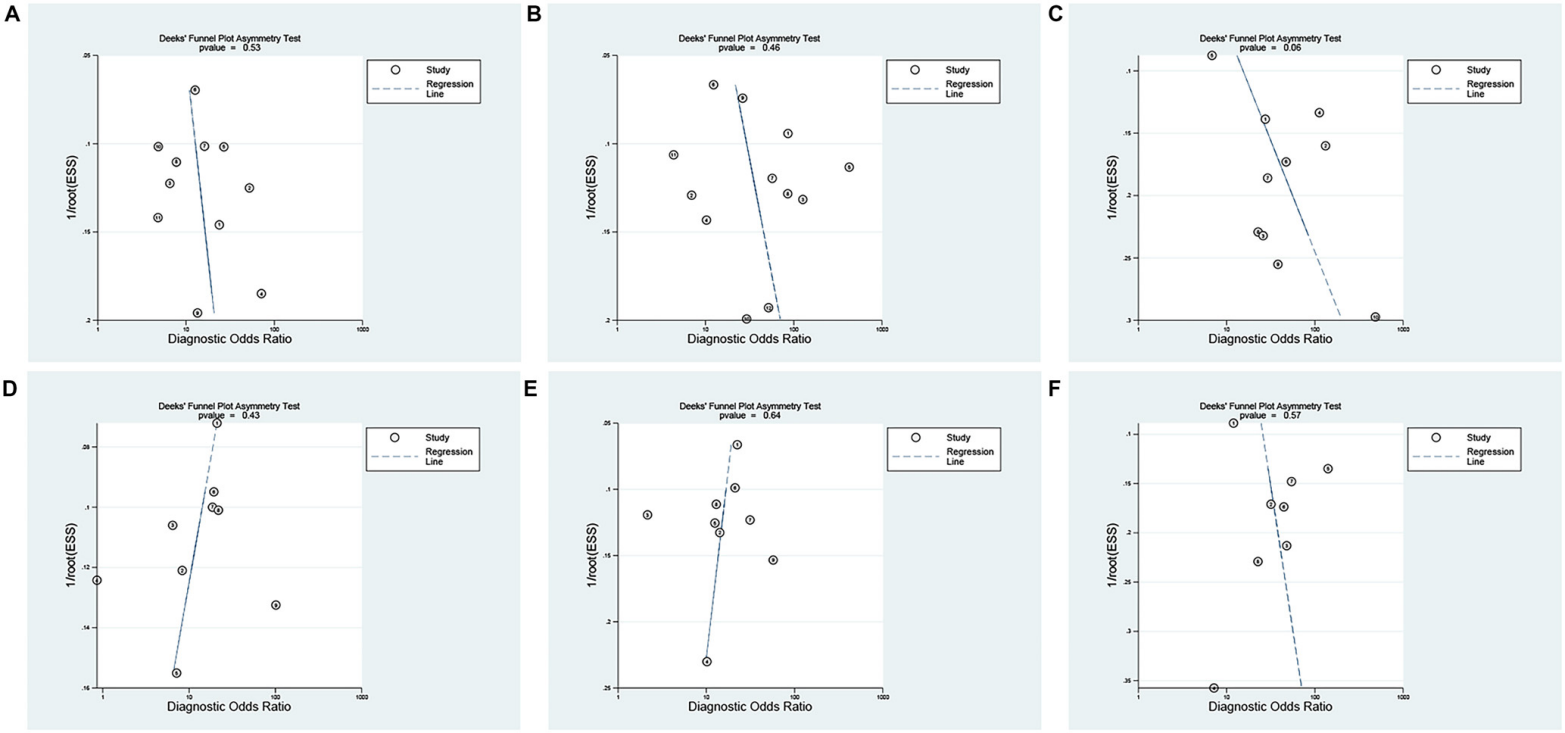
**Publication bias for SWE.** (A) Fibrosis (*F* ≥ 2) using pSWE; (B) Advanced fibrosis (F ≥ 3) using pSWE; (C) Cirrhosis (*F* ═ 4) using pSWE; (D) Fibrosis (*F* ≥ 2) using 2-D SWE; (E) Advanced fibrosis (*F* ≥ 3) using 2-D SWE; (F) Cirrhosis (*F* ═ 4) using 2-D SWE. SWE: Shear wave elastography; pSWE: Point shear wave elastography; 2-D SWE: Two-dimensional shear wave elastography.

To assess potential reporting bias within each study group, the Deeks test was performed. The results of pSWE and 2-D SWE for identifying significant fibrosis, advanced fibrosis, and cirrhosis showed no evidence of publication bias based on the analysis (Figure 5).

## Discussion

The histological spectrum of MASLD spans from simple steatosis to MASH, severe fibrosis, and potential cirrhosis. Research has demonstrated that advanced fibrosis and cirrhosis are significant predictors of mortality and liver-related complications [31]. Detecting fibrosis at *F* ≥ 2 is clinically crucial for managing MASLD patients, as this stage often progresses to more severe disease, including cirrhosis, leading to substantial morbidity and mortality [11]. Accurate staging of liver fibrosis in patients is therefore essential. Ultrasound elastography offers a non-invasive, accessible method for assessing liver fibrosis. It helps predict disease progression and guide treatment strategies. To evaluate the diagnostic performance of pSWE and 2-D SWE in identifying liver fibrosis non-invasively in MASLD patients, we conducted a meta-analysis of 20 studies comprising 2241 patients. Using a bivariate mixed-effects model, we calculated sensitivity, specificity, PLR, NLR, DOR, and AUC for pSWE and 2-D SWE across various fibrosis stages. Our findings revealed that both pSWE and 2-D SWE demonstrated strong diagnostic accuracy for staging advanced fibrosis and cirrhosis. For pSWE, the AUC was 0.91 for *F* ≥ 3 and 0.94 for *F* ═ 4. Additionally, pSWE showed favorable diagnostic accuracy for significant fibrosis (*F* ≥ 2), achieving an AUC of 0.84. Importantly, there was no significant difference in performance between the two techniques across fibrosis stages. The study also highlighted pSWE’s effectiveness in detecting significant fibrosis, consistent with findings from Jiang et al. [32], who analyzed nine studies (982 patients). Both studies reported similar AUC values (0.84 vs 0.86 for *F* ≥ 2, 0.91 vs 0.94 for *F* ≥ 3, and 0.94 vs 0.95 for *F* ═ 4) and specificity values (0.83 vs 0.84, 0.87 vs 0.88, and 0.91 vs 0.91). However, Jiang et al. reported higher sensitivity for advanced fibrosis and cirrhosis, potentially due to differences in study inclusion criteria.

We excluded studies with a 6-month gap between pSWE and biopsy measurements and incorporated more recent research beyond Jiang et al.’s 2018 cutoff. This broader inclusion may provide a more comprehensive perspective. Similarly, a meta-analysis by Selvaraj et al. [33] evaluated 11 pSWE studies, reporting AUCs of 0.86 (95% CI 0.78–0.90) for significant fibrosis, 0.89 (95% CI 0.83–0.95) for advanced fibrosis, and 0.90 (95% CI 0.82–0.95) for cirrhosis. Their specificity results—0.85 (95% CI 0.80–0.98), 0.86 (95% CI 0.82–0.92), and 0.88 (95% CI 0.82–0.92)—were also similar to ours. However, their sensitivity values were lower (0.69, 0.80, and 0.76, respectively). This discrepancy may stem from their focus on studies utilizing virtual touch quantification (VTQ) on the Siemens platform, whereas our analysis included ElastPQ® technology. While both VTQ and ElastPQ are ultrasonic elastography techniques, they differ fundamentally in technology [34, 35]. By including a wider range of pSWE technologies and updated research data, our study provided a more comprehensive assessment. Regarding 2-D SWE, our meta-analysis revealed AUCs of 0.83 for *F* ≥ 2, 0.85 for *F* ≥ 3, and 0.90 for *F* ═ 4. In comparison, a previous individual patient data meta-analysis [36] reported slightly higher AUCs (0.86, 0.93, and 0.92, respectively) for diagnosing significant fibrosis, severe fibrosis, and cirrhosis. This discrepancy may be explained by differences in methodologies. The prior study included only 156 MASLD patients and did not standardize cutoff values, whereas we included diverse 2-D SWE systems (e.g., Aplio i800, LOGIQ E9, and Acuson S3000) and adhered to unified inclusion and exclusion criteria. Selvaraj et al. [33] also conducted a 2-D SWE meta-analysis of four studies, reporting lower AUCs (0.75, 0.72, and 0.88 for *F* ≥ 2, *F* ≥ 3, and *F* ═ 4, respectively) and sensitivity values (0.71, 0.72, and 0.78, respectively). The differences may be attributed to their reliance on studies published before 2021, while our analysis incorporated newer studies. For instance, Ogino et al. [37] (2023) used LOGIQ E9 to assess 107 MASLD patients, reporting AUCs of 0.87 and 0.92 for significant fibrosis and cirrhosis, respectively. Similarly, Seo et al. [24] reported AUCs of 0.90 and 0.92 for these stages using 2-D SWE. Including such recent studies enhanced the robustness of our evaluation. In summary, both pSWE and 2-D SWE provide reliable diagnostic tools for staging liver fibrosis in MASLD patients. However, our analysis indicates that pSWE may offer slightly superior sensitivity and diagnostic performance across fibrosis stages, while incorporating more recent studies and broader elastography technologies contributes to a more comprehensive understanding of diagnostic accuracy.

Based on current research findings on SWE, several factors may affect its clinical application. Firstly, the detection accuracy of pSWE and 2-D SWE is closely tied to the operator’s technique. As these technologies become more widely adopted, their performance in diagnosing liver fibrosis stages is expected to improve. However, confounding factors, such as human error and equipment malfunctions highlight the need for further research to optimize their clinical use and establish consensus-driven standards. Notably, previous studies have shown that pSWE measurements are influenced by the operator’s experience, which can result in both inter-observer and intra-observer variability [38]. Additionally, 2-D SWE may occasionally fail to provide valid measurement results [39]. To explore the sources of variability, we conducted subgroup analyses across various dimensions, as shown in Tables 2 and 3. These covariates included the region of enrolled patients (Asia vs non-Asia), the system used (Aixplorer f vs non-Aixplorer f), sex (male proportion >50% vs male proportion <50%), and clinical setting (multicenter vs single center). Findings in 2-D SWE Subgroup Analysis: Across all fibrosis stages (*F* ≥ 2, *F* ≥ 3, and *F* ═ 4), diagnostic specificity in Asian regions was lower than in non-Asian regions. At the *F* ≥ 2 stage, studies using the Aixplorer device showed lower sensitivity and specificity compared to other devices. For stages *F* ≥ 3 and *F* ═ 4, the Aixplorer device demonstrated lower specificity than other systems. At stages *F* ≥ 2 and *F* ≥ 3, diagnostic sensitivity was lower in populations with a male proportion >50% compared to those with a male proportion < 50%. Multicenter settings demonstrated lower diagnostic sensitivity than single-center settings for stages *F* ≥ 3 and *F* ═ 4. At the *F* ═ 4 stage, specificity was lower in obese populations than in non-obese populations. Findings in pSWE Subgroup Analysis: Across all stages (*F* ≥ 2, *F* ≥ 3, and *F* ═ 4), diagnostic specificity was lower in Asian regions compared to non-Asian regions. At the *F* ≥ 2 stage, sensitivity in Asian regions was also lower than in non-Asian regions. For the *F* ═ 4 stage, studies using the Acuson device showed lower specificity than those using other devices. At stages *F* ≥ 2 and *F* ≥ 3, diagnostic sensitivity was lower in populations with a male proportion >50% compared to those with a male proportion <50%. At the *F* ═ 4 stage, diagnostic specificity was also lower in populations with a male proportion >50% compared to those with a male proportion <50%. Across all stages, multicenter settings demonstrated lower diagnostic sensitivity than single-center settings. These results suggest that variations in clinical systems, patient demographics, and settings may contribute to the observed heterogeneity. From a clinical perspective, it would be beneficial to emphasize studies that involve diverse systems and to develop standardized guidelines for their use. Furthermore, the findings suggest that diagnostic sensitivity may be higher in female populations, highlighting the importance of considering sex when selecting patient populations in clinical practice. Comparison of pSWE and 2-D SWE: Our study also compared the synthesized sensitivity of pSWE and 2-D SWE. For detecting significant fibrosis and advanced fibrosis stages, the synthesized sensitivity of pSWE was slightly lower than that of 2-D SWE (0.78 vs 0.89), though this difference was not statistically significant. Similarly, in detecting cirrhosis, the synthesized sensitivity of pSWE was slightly higher than that of 2-D SWE (0.81 vs 0.92), but again, the difference was not significant. These findings indicate that both methods exhibit similar diagnostic performance and are effective for identifying varying degrees of fibrosis. However, preferences may vary depending on the fibrosis stage. Additional high-quality studies directly comparing these methods within the same population are needed for more in-depth insights.

*Limitations of the Study:* Our study assessed the diagnostic performance of two non-invasive methods, pSWE and 2-D SWE, in MASLD patients. It also compared their sensitivity differences within the same population, providing up-to-date and comprehensive evidence for clinical application. However, several limitations warrant consideration: Optimal cutoff values: Current equipment and usage lack consensus on optimal cutoff values. Variability in cutoff values used across systems may have contributed to result heterogeneity. Limited direct comparisons: While this study included a sensitivity comparison of pSWE and 2-D SWE, only two studies directly assessed the same patient populations. This limits our ability to confirm which method is superior for evaluating different fibrosis stages. Language bias: The inclusion of only English-language studies may have introduced selection bias. In conclusion, while pSWE and 2-D SWE show comparable diagnostic performance, further research is needed to refine their application, develop standardized guidelines, and address gaps in the literature, such as optimal cutoff values and population-specific variations.

## Conclusion

Both pSWE and 2-D SWE demonstrated satisfactory sensitivity and specificity in distinguishing different stages of liver fibrosis, making them suitable for clinical application. Specifically, 2-D SWE exhibited greater sensitivity than pSWE in detecting significant and advanced fibrosis, while pSWE showed higher sensitivity than 2-D SWE in identifying cirrhosis. However, these differences were not statistically significant. Future research is needed to confirm and standardize cutoff values and usage protocols for both pSWE and 2-D SWE. Moreover, more direct comparisons between the two techniques are essential to determine the superior method for evaluating various stages of fibrosis.

## Supplemental data

Supplemental data are available at the following link: https://bjbms.org/ojs/index.php/bjbms/article/view/11577/3698.

## Data Availability

The data supporting the findings of this study are available from the corresponding author upon reasonable request.
